# Tris(ethyl­enediamine)cobalt(II) sulfate

**DOI:** 10.1107/S1600536810016168

**Published:** 2010-05-08

**Authors:** Bunlawee Yotnoi, Athittaya Seeharaj, Yothin Chimupala, Apinpus Rujiwatra

**Affiliations:** a Department of Chemistry, Faculty of Science, Chiang Mai University, Chiang Mai 50200, Thailand

## Abstract

The structure of the title compound, [Co^II^(C_2_H_8_N_2_)_3_]SO_4_, the cobalt example of [*M*(C_2_H_8_N_2_)_3_]SO_4_, is reported. The Co and S atoms are located at the 2*d* and 2*c* Wyckoff sites (point symmetry 32), respectively. The Co atom is coordinated by six N atoms of three chelating ethyl­enediamine mol­ecules generated from half of the ethyl­enediamine mol­ecule in the asymmetric unit. The O atoms of the sulfate anion are disordered mostly over two crystallographic sites. The third disorder site of O (site symmetry 3) has a site occupancy approaching zero. The H atoms of the ethyl­enediamine mol­ecules inter­act with the sulfate anions *via* inter­molecular N—H⋯O hydrogen-bonding inter­actions.

## Related literature

For isostructural [*M*(C_2_H_8_N_2_)_3_]SO_4_ complexes, see: Haque *et al.* (1970 ▶); Cullen & Lingafelter (1970 ▶); Daniels *et al.* (1995 ▶); Lu (2009 ▶) for the nickel, copper, vanadium and manganese analogues, respectively.
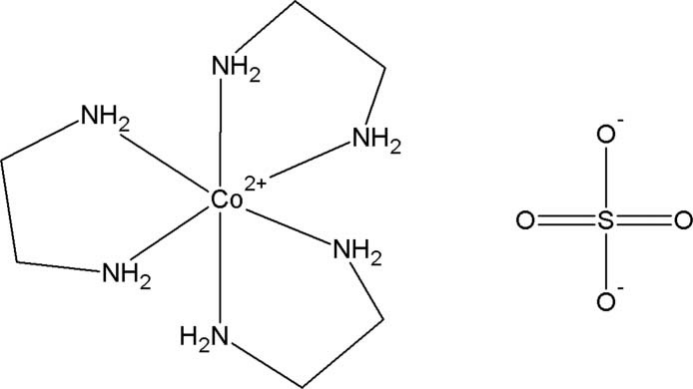


## Experimental

### 

#### Crystal data

[Co(C_2_H_8_N_2_)_3_]SO_4_
*M* *_r_* = 335.30
Trigonal, 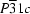

*a* = 8.9920 (2) Å
*c* = 9.5927 (3) Å
*V* = 671.71 (3) Å^3^
*Z* = 2
Mo *K*α radiation
μ = 1.45 mm^−1^
*T* = 298 K
0.48 × 0.22 × 0.20 mm

#### Data collection

Bruker SMART CCD area-detector diffractometer
Absorption correction: multi-scan (*SADABS*; Sheldrick, 1996 ▶) *T*_min_ = 0.543, *T*_max_ = 0.760
3638 measured reflections
688 independent reflections
589 reflections with *I* > 2σ(*I*)
*R*_int_ = 0.027

#### Refinement

*R*[*F* ^2^ > 2σ(*F* ^2^)] = 0.028
*wR*(*F* ^2^) = 0.069
*S* = 1.06
688 reflections
47 parameters
16 restraints
H-atom parameters constrained
Δρ_max_ = 0.25 e Å^−3^
Δρ_min_ = −0.29 e Å^−3^

### 

Data collection: *SMART* (Bruker, 2003 ▶); cell refinement: *SAINT* (Bruker, 2003 ▶); data reduction: *SAINT*; program(s) used to solve structure: *SHELXS97* (Sheldrick, 2008 ▶) and *WinGX* (Farrugia, 1999 ▶); program(s) used to refine structure: *SHELXL97* (Sheldrick, 2008 ▶) and *WinGX* (Farrugia, 1999 ▶); molecular graphics: *DIAMOND* (Brandenburg, 2006 ▶); software used to prepare material for publication: *publCIF* (Westrip, 2010 ▶).

## Supplementary Material

Crystal structure: contains datablocks I, global. DOI: 10.1107/S1600536810016168/tk2667sup1.cif

Structure factors: contains datablocks I. DOI: 10.1107/S1600536810016168/tk2667Isup2.hkl

Additional supplementary materials:  crystallographic information; 3D view; checkCIF report

## Figures and Tables

**Table 1:** Hydrogen-bond geometry (Å, °)

*D*—H⋯*A*	*D*—H	H⋯*A*	*D*⋯*A*	*D*—H⋯*A*
N1—H1*A*⋯O3^i^	0.90	2.13	2.889 (12)	142
N1—H1*A*⋯O1^i^	0.90	2.15	3.049 (7)	176
N1—H1*A*⋯O2^ii^	0.90	2.22	3.054 (8)	155
N1—H1*A*⋯O2^iii^	0.90	2.32	3.104 (11)	145
N1—H1*B*⋯O2^iv^	0.90	1.98	2.843 (6)	161
N1—H1*B*⋯O1	0.90	2.48	3.353 (14)	165
N1—H1*B*⋯O1^v^	0.90	2.52	3.256 (10)	139

Symmetry codes: (i) 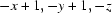; (ii) 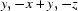; (iii) 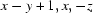; (iv) 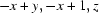; (v) 
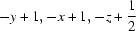
.

**Fig. 1.**
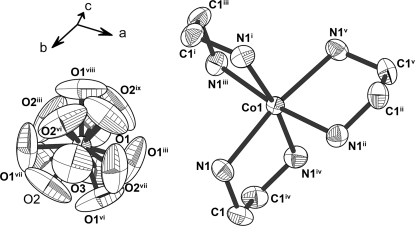
View of the title complex showing atom numbering scheme. Displacement ellipsoids for non-H atoms are drawn at the 50% probability level. [Symmetry codes: (i) -y+1, x-y, z; (ii) -x+y+1, -x+1, z; (iii) -y+1, -x+1, -z+1/2; (iv) -x+y+1, y, -z+1/2; (v) x, x-y, -z+1/2; (vi) -y+1, x-y+1, z, (vii) -x+y, -x+1, z; (viii) -x+y, y, -z+1/2; (ix) x, x-y+1, -z+1/2]. Hydrogen atoms are omitted.

**Fig. 2.**
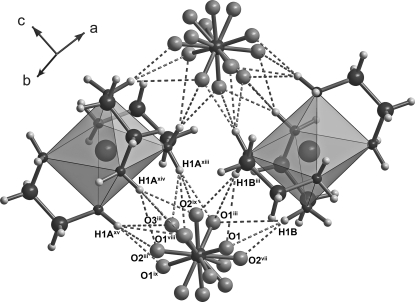
View of the hydrogen bonding interactions (dotted lines) between the disordered sulfate O atoms and the amino-H atoms of the [CoII(C2H8N2)]2+ cations. [Symmetry codes: (ii) -x+y+1, -x+1, z; (iii) -y+1, -x+1, -z+1/2; (viii) -x+y, y, -z+1/2; (ix) x, x-y+1, -z+1/2; (xiii) y, x, z+1/2; (xiv) -y+x, -y+1, -z+1/2; (xv) -x+1, -x+y+1, z+1/2].

## supplementary crystallographic information

### Comment

The title complex, [Co^II^(C_2_H_8_N_2_)_3_]SO_4_ (Fig. 1), is isostructural to the earlier reported [Ni^II^(C_2_H_8_N_2_)_3_]SO_4_ (Haque *et al.*,, 1970), [V^II^(C_2_H_8_N_2_)_3_]SO_4_ (Daniels *et al.*, 1995), [Mn^II^(C_2_H_8_N_2_)_3_]SO_4_ (Lu, 2009) and [Cu^II^(C_2_H_8_N_2_)_3_]SO_4_ (Cullen & Lingafelter, 1970) complexes, constituting the [M^II^(C_2_H_2_N_2_)_3_]SO_4_ series. The [M^II^(C_2_H_2_N_2_)_3_]SO_4_ structures crystallize in the same trigonal space group of P31c with quite similar cell parameters. Likewise, the metal and sulfur atoms are positioned in the same crystallographic sites; M^II^ on the 2*d* and S on the 2*c *Wyckoff sites (each with point symmetry 32). The disorder about the six-fold rotation axis found in the sulfate anion is intriguingly common in each structure, although the number of unique O atoms varies from two to four. In the structure of [Co^II^(C_2_H_8_N_2_)_3_]SO_4_, the O atoms were refined as being disordered over three crystallographic sites, although the site occupancy of O3 located on the 4*f *Wyckoff site approaches zero. The bond length associated with this O3 atom (S1—O3; 1.382 (16) Å) is notably shorter than the other S—O bonds (1.431 (5)–1.445 (5) Å). The disordered sulfate anions are linked to the [Co^II^(C_2_H_8_N_2_)_3_]^2+^ cations by hydrogen bonding interactions of N—H···O type to form a hydrogen-bonding supramolecular network. The hydrogen bonding geometries are consistent with those of the previously reported [M^II^(C_2_H_2_N_2_)_3_]SO_4_ complexes.

### Experimental

Orange blocks of the title complex were synthesized and grown from the sovolthermal reaction of Co(NO_3_)_2_.6H_2_O (1.34 mmol), NH_2_SO_3_H (1.34 mmol), NH_2_C_2_H_4_NH_2_ (3.89 mmol) in ethylene glycol (160 mmol), conducted at 453 K for 72 h.

### Refinement

The O atoms were positioned from a difference Fourier map, and refined with restraints using commands SUMP, SADI and SIMU in *SHELXL* (Sheldrick, 2008). Although there was an indication for further splitting of the O2 atom, after the final cycles of refinement, such action did not give a better result. All H-atoms were treated as riding groups on the bonded atoms, with C—H = 0.97 Å and N—H 0.90 Å, and with U_iso_(H) = 1.2U_equiv_(C, N).

### Crystal data

**Table:**

[Co(C_2_H_8_N_2_)_3_]SO_4_	*D*_x_ = 1.658 Mg m^−^^3^
*M**_r_* = 335.30	Mo *K*α radiation, λ = 0.71073 Å
Trigonal, *P*31*c*	Cell parameters from 589 reflections
Hall symbol: -P 3 2c	θ = 2.6–31.0°
*a* = 8.9920 (2) Å	µ = 1.45 mm^−^^1^
*c* = 9.5927 (3) Å	*T* = 298 K
*V* = 671.71 (3) Å^3^	Block, orange
*Z* = 2	0.48 × 0.22 × 0.20 mm
*F*(000) = 354	

### Data collection

**Table:**

Bruker SMART CCD area-detector diffractometer	688 independent reflections
Radiation source: fine-focus sealed tube	589 reflections with *I* > 2σ(*I*)
graphite	*R*_int_ = 0.027
? scan	θ_max_ = 31.0°, θ_min_ = 2.6°
Absorption correction: multi-scan (*SADABS*; Sheldrick, 1996)	*h* = −8→10
*T*_min_ = 0.543, *T*_max_ = 0.760	*k* = −11→11
3638 measured reflections	*l* = −11→13

### Refinement

**Table:**

Refinement on *F*^2^	Primary atom site location: structure-invariant direct methods
Least-squares matrix: full	Secondary atom site location: difference Fourier map
*R*[*F*^2^ > 2σ(*F*^2^)] = 0.028	Hydrogen site location: inferred from neighbouring sites
*wR*(*F*^2^) = 0.069	H-atom parameters constrained
*S* = 1.06	*w* = 1/[σ^2^(*F*_o_^2^) + (0.0354*P*)^2^ + 0.1217*P*] where *P* = (*F*_o_^2^ + 2*F*_c_^2^)/3
688 reflections	(Δ/σ)_max_ < 0.001
47 parameters	Δρ_max_ = 0.25 e Å^−^^3^
16 restraints	Δρ_min_ = −0.29 e Å^−^^3^

### Special details

**Table:**

Geometry. All esds (except the esd in the dihedral angle between two l.s. planes) are estimated using the full covariance matrix. The cell esds are taken into account individually in the estimation of esds in distances, angles and torsion angles; correlations between esds in cell parameters are only used when they are defined by crystal symmetry. An approximate (isotropic) treatment of cell esds is used for estimating esds involving l.s. planes.
Refinement. Refinement of *F*^2^ against ALL reflections. The weighted *R*-factor wR and goodness of fit *S* are based on *F*^2^, conventional *R*-factors *R* are based on *F*, with *F* set to zero for negative *F*^2^. The threshold expression of *F*^2^ > σ(*F*^2^) is used only for calculating *R*-factors(gt) etc. and is not relevant to the choice of reflections for refinement. *R*-factors based on *F*^2^ are statistically about twice as large as those based on *F*, and *R*- factors based on ALL data will be even larger.

### Fractional atomic coordinates and isotropic or equivalent isotropic displacement parameters (Å^2^)

**Table:**

	*x*	*y*	*z*	*U*_iso_*/*U*_eq_	Occ. (<1)
Co1	0.6667	0.3333	0.2500	0.02175 (16)	
N1	0.68784 (18)	0.54599 (18)	0.12760 (13)	0.0332 (3)	
H1A	0.6936	0.5265	0.0363	0.040*	
H1B	0.5954	0.5579	0.1418	0.040*	
S1	0.3333	0.6667	0.2500	0.0240 (2)	
C1	0.8446 (2)	0.7024 (2)	0.17145 (19)	0.0388 (4)	
H1C	0.8405	0.8031	0.1409	0.047*	
H1D	0.9444	0.7056	0.1297	0.047*	
O1	0.3029 (19)	0.5088 (9)	0.1852 (8)	0.096 (3)	0.319 (8)
O2	0.339 (2)	0.7851 (9)	0.1475 (6)	0.096 (4)	0.316 (9)
O3	0.3333	0.6667	0.1059 (16)	0.086 (8)	0.094 (10)

### Atomic displacement parameters (Å^2^)

**Table:**

	*U*^11^	*U*^22^	*U*^33^	*U*^12^	*U*^13^	*U*^23^
Co1	0.0226 (2)	0.0226 (2)	0.0201 (2)	0.01129 (10)	0.000	0.000
N1	0.0410 (8)	0.0330 (7)	0.0283 (7)	0.0204 (6)	−0.0033 (5)	0.0031 (5)
S1	0.0243 (3)	0.0243 (3)	0.0233 (4)	0.01215 (14)	0.000	0.000
C1	0.0445 (10)	0.0267 (8)	0.0413 (9)	0.0147 (7)	0.0058 (7)	0.0076 (6)
O1	0.185 (9)	0.051 (4)	0.063 (4)	0.069 (5)	−0.012 (5)	−0.016 (3)
O2	0.194 (12)	0.055 (4)	0.044 (3)	0.067 (5)	−0.012 (4)	0.012 (3)
O3	0.118 (11)	0.118 (11)	0.021 (11)	0.059 (5)	0.000	0.000

### Geometric parameters (Å, °)

**Table:**

Co1—N1^i^	2.1696 (13)	S1—O2^vi^	1.431 (5)
Co1—N1^ii^	2.1696 (13)	S1—O2^v^	1.431 (5)
Co1—N1^iii^	2.1696 (13)	S1—O2^vii^	1.431 (5)
Co1—N1^iv^	2.1696 (13)	S1—O2^viii^	1.431 (5)
Co1—N1	2.1696 (13)	S1—O2^ix^	1.431 (5)
Co1—N1^v^	2.1696 (13)	S1—O1^ix^	1.445 (5)
N1—C1	1.469 (2)	S1—O1^viii^	1.445 (5)
N1—H1A	0.9000	S1—O1^vi^	1.445 (5)
N1—H1B	0.9000	S1—O1^vii^	1.445 (5)
S1—O3	1.382 (16)	C1—C1^iv^	1.512 (4)
S1—O3^v^	1.382 (16)	C1—H1C	0.9700
S1—O2	1.431 (5)	C1—H1D	0.9700
			
N1^i^—Co1—N1^ii^	80.49 (7)	O2^viii^—S1—O1^viii^	110.7 (4)
N1^i^—Co1—N1^iii^	93.48 (5)	O2^ix^—S1—O1^viii^	138.0 (11)
N1^ii^—Co1—N1^iii^	93.17 (8)	O1^ix^—S1—O1^viii^	63.4 (8)
N1^i^—Co1—N1^iv^	93.17 (8)	O3—S1—O1^vi^	64.5 (3)
N1^ii^—Co1—N1^iv^	93.48 (5)	O3^v^—S1—O1^vi^	115.5 (3)
N1^iii^—Co1—N1^iv^	171.28 (7)	O2—S1—O1^vi^	57.2 (5)
N1^i^—Co1—N1	93.48 (5)	O2^vi^—S1—O1^vi^	110.7 (4)
N1^ii^—Co1—N1	171.28 (8)	O2^v^—S1—O1^vi^	138.0 (11)
N1^iii^—Co1—N1	93.48 (5)	O2^vii^—S1—O1^vi^	69.9 (6)
N1^iv^—Co1—N1	80.49 (7)	O2^viii^—S1—O1^vi^	45.7 (4)
N1^i^—Co1—N1^v^	171.28 (8)	O2^ix^—S1—O1^vi^	119.2 (10)
N1^ii^—Co1—N1^v^	93.48 (5)	O1^ix^—S1—O1^vi^	93.3 (11)
N1^iii^—Co1—N1^v^	80.49 (7)	O1^viii^—S1—O1^vi^	102.9 (4)
N1^iv^—Co1—N1^v^	93.48 (5)	O3—S1—O1^vii^	115.5 (3)
N1—Co1—N1^v^	93.17 (8)	O3^v^—S1—O1^vii^	64.5 (3)
C1—N1—Co1	107.94 (10)	O2—S1—O1^vii^	138.0 (11)
C1—N1—H1A	110.1	O2^vi^—S1—O1^vii^	69.9 (6)
Co1—N1—H1A	110.1	O2^v^—S1—O1^vii^	57.2 (5)
C1—N1—H1B	110.1	O2^vii^—S1—O1^vii^	110.7 (4)
Co1—N1—H1B	110.1	O2^viii^—S1—O1^vii^	119.2 (10)
H1A—N1—H1B	108.4	O2^ix^—S1—O1^vii^	45.7 (4)
O3—S1—O3^v^	180.000 (3)	O1^ix^—S1—O1^vii^	102.9 (4)
O3—S1—O2	46.6 (3)	O1^viii^—S1—O1^vii^	93.3 (11)
O3^v^—S1—O2	133.4 (3)	O1^vi^—S1—O1^vii^	161.1 (12)
O3—S1—O2^vi^	46.6 (3)	N1—C1—C1^iv^	108.84 (12)
O3^v^—S1—O2^vi^	133.4 (3)	N1—C1—H1C	109.9
O2—S1—O2^vi^	78.0 (5)	C1^iv^—C1—H1C	109.9
O3—S1—O2^v^	133.4 (3)	N1—C1—H1D	109.9
O3^v^—S1—O2^v^	46.6 (3)	C1^iv^—C1—H1D	109.9
O2—S1—O2^v^	104.4 (11)	H1C—C1—H1D	108.3
O2^vi^—S1—O2^v^	99.7 (7)	O2^vi^—O1—O2^viii^	91.9 (8)
O3—S1—O2^vii^	133.4 (3)	O2^vi^—O1—S1	66.5 (5)
O3^v^—S1—O2^vii^	46.6 (3)	O2^viii^—O1—S1	60.9 (3)
O2—S1—O2^vii^	99.7 (7)	O2^vi^—O1—O1^vii^	75.7 (11)
O2^vi^—S1—O2^vii^	176.3 (13)	O2^viii^—O1—O1^vii^	117.8 (4)
O2^v^—S1—O2^vii^	78.0 (5)	S1—O1—O1^vii^	58.3 (4)
O3—S1—O2^viii^	46.6 (3)	O2^vi^—O1—O2^ix^	108.3 (7)
O3^v^—S1—O2^viii^	133.4 (3)	O2^viii^—O1—O2^ix^	92.2 (8)
O2—S1—O2^viii^	78.0 (5)	S1—O1—O2^ix^	54.6 (4)
O2^vi^—S1—O2^viii^	78.0 (5)	O1^viii^—O2—O1^vi^	129.8 (7)
O2^v^—S1—O2^viii^	176.3 (13)	O1^viii^—O2—S1	67.8 (4)
O2^vii^—S1—O2^viii^	104.4 (11)	O1^vi^—O2—S1	61.9 (4)
O3—S1—O2^ix^	133.4 (3)	O1^viii^—O2—O1^ix^	63.3 (12)
O3^v^—S1—O2^ix^	46.6 (3)	O1^vi^—O2—O1^ix^	87.5 (9)
O2—S1—O2^ix^	176.3 (13)	S1—O2—O1^ix^	55.5 (3)
O2^vi^—S1—O2^ix^	104.4 (11)	O1^viii^—O2—O2^vi^	49.8 (6)
O2^v^—S1—O2^ix^	78.0 (5)	O1^vi^—O2—O2^vi^	95.3 (5)
O2^vii^—S1—O2^ix^	78.0 (5)	S1—O2—O2^vi^	51.0 (2)
O2^viii^—S1—O2^ix^	99.7 (7)	O1^ix^—O2—O2^vi^	91.9 (8)
O3—S1—O1^ix^	115.5 (3)	O1^viii^—O2—O2^viii^	106.1 (5)
O3^v^—S1—O1^ix^	64.5 (3)	S1—O2—O2^viii^	51.0 (2)
O2—S1—O1^ix^	69.9 (6)	O1^ix^—O2—O2^viii^	102.2 (4)
O2^vi^—S1—O1^ix^	119.2 (10)	O2^vi^—O2—O2^viii^	60.000 (1)
O2^v^—S1—O1^ix^	45.7 (4)	O2^vi^—O3—O2^viii^	107.9 (8)
O2^vii^—S1—O1^ix^	57.2 (5)	O2^vi^—O3—S1	69.0 (8)
O2^viii^—S1—O1^ix^	138.0 (11)	O2^viii^—O3—S1	69.0 (8)
O2^ix^—S1—O1^ix^	110.7 (4)	O2^vi^—O3—O1^viii^	61.1 (6)
O3—S1—O1^viii^	64.5 (3)	O2^viii^—O3—O1^viii^	128.2 (13)
O3^v^—S1—O1^viii^	115.5 (3)	S1—O3—O1^viii^	59.8 (5)
O2—S1—O1^viii^	45.7 (4)	O2^vi^—O3—O1^vi^	128.2 (13)
O2^vi^—S1—O1^viii^	57.2 (5)	O2^viii^—O3—O1^vi^	47.4 (6)
O2^v^—S1—O1^viii^	69.9 (6)	S1—O3—O1^vi^	59.8 (5)
O2^vii^—S1—O1^viii^	119.2 (10)	O1^viii^—O3—O1^vi^	96.9 (7)

Symmetry codes: (i) −*x*+*y*+1, −*x*+1, *z*; (ii) *x*, *x*−*y*, −*z*+1/2; (iii) −*y*+1, *x*−*y*, *z*; (iv) −*x*+*y*+1, *y*, −*z*+1/2; (v) −*y*+1, −*x*+1, −*z*+1/2; (vi) −*y*+1, *x*−*y*+1, *z*; (vii) −*x*+*y*, *y*, −*z*+1/2; (viii) −*x*+*y*, −*x*+1, *z*; (ix) *x*, *x*−*y*+1, −*z*+1/2.

### Hydrogen-bond geometry (Å, °)

**Table:**

*D*—H···*A*	*D*—H	H···*A*	*D*···*A*	*D*—H···*A*
N1—H1A···O3^x^	0.90	2.13	2.889 (12)	142
N1—H1A···O1^x^	0.90	2.15	3.049 (7)	176
N1—H1A···O2^xi^	0.90	2.22	3.054 (8)	155
N1—H1A···O2^xii^	0.90	2.32	3.104 (11)	145
N1—H1B···O2^viii^	0.90	1.98	2.843 (6)	161
N1—H1B···O1	0.90	2.48	3.353 (14)	165
N1—H1B···O1^v^	0.90	2.52	3.256 (10)	139

Symmetry codes: (x) −*x*+1, −*y*+1, −*z*; (xi) *y*, −*x*+*y*, −*z*; (xii) *x*−*y*+1, *x*, −*z*; (viii) −*x*+*y*, −*x*+1, *z*; (v) −*y*+1, −*x*+1, −*z*+1/2.
